# Supplementary figures and images for: Can post-capture photographic identification as a wildlife marking technique be undermined by observer error? A case study using King Cobras in northeast Thailand
Source: PLoS One. 2020 Dec 9;15(12):e0242826. doi: 10.1371/journal.pone.0242826 (PMC7725303; doi:10.1371/journal.pone.0242826)

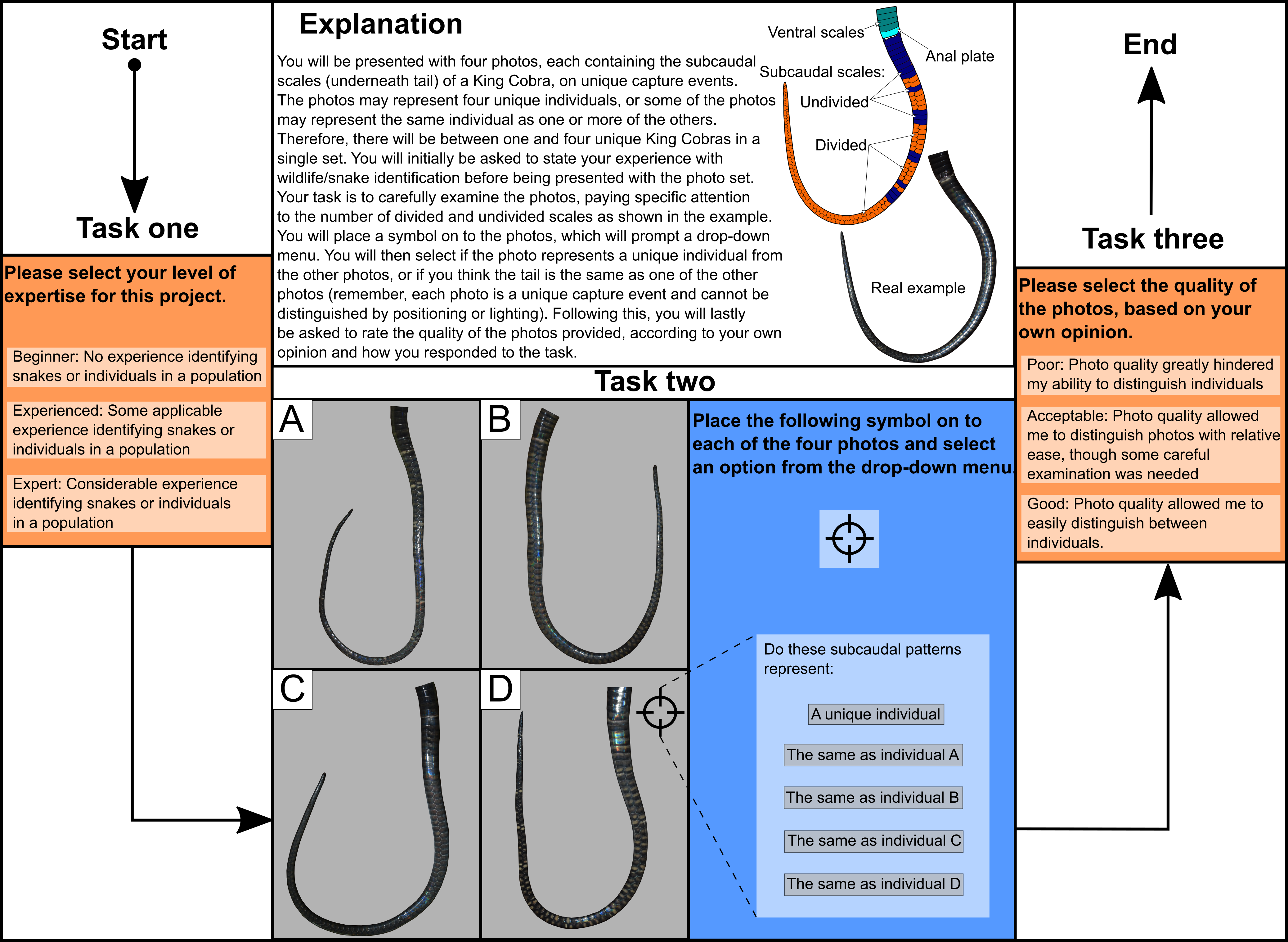

Supplement: S1 Fig — A preliminary design for the subcaudal scale arrangement project workflow as seen by Zooniverse volunteers. (TIF) [file pone.0242826.s001.tif]
